# Effect of Resin Type, Layer Thickness, and Printing Orientation on the Mechanical and Surface Properties of 3D-Printed Occlusal Splints

**DOI:** 10.3390/polym18020290

**Published:** 2026-01-21

**Authors:** Beyza Tandogan, Faruk Emir, Gulsum Ceylan

**Affiliations:** 1 Graduate School of Health Sciences, Department of Prosthodontics, Istanbul Medipol University, Fatih 34810, Istanbul, Turkey; 2 Department of Prosthodontics, Gülhane Faculty of Dentistry, Health Sciences University, Ankara 06010, Turkey; 3 Department of Prosthodontics, School of Dentistry, Istanbul Medipol University, Ataturk Bulvari, No:27, Unkapani, Fatih 34083, Istanbul, Turkey

**Keywords:** digital light processing, layer thickness, occlusal splint, printing orientation, resin materials, 3D printing

## Abstract

This in vitro study aimed to evaluate the effects of resin type, layer thickness, and printing orientation on the surface and mechanical properties of 3D-printed occlusal splints fabricated using digital light processing (DLP) technology. Three commercially available splint resins (KeySplint Hard, Freeprint Splint 2.0, and V-Print Splint) were used to fabricate 180 rectangular specimens with two-layer thicknesses (50 µm and 100 µm) and three printing orientations (0°, 45°, 90°). Surface roughness (*Ra*, *Rz*), gloss, microhardness, flexural strength, and elastic modulus were measured. Statistical analysis was performed using robust ANOVA with Bonferroni correction. Resin type and printing orientation significantly influenced all surface and mechanical properties (*p* < 0.001), while layer thickness had a limited effect. Keystone resin exhibited the smoothest surface and highest gloss, whereas Freeprint resin showed the highest microhardness and elastic modulus. Printing at 45° generally enhanced flexural strength and provided more balanced mechanical performance. SEM analysis confirmed that surface morphology varied with orientation, correlating with profilometric and gloss measurements. Resin composition and printing orientation are critical determinants of the mechanical and surface performance of 3D-printed occlusal splints. Optimizing these parameters can improve durability, esthetics, and clinical functionality. All tested materials achieved clinically acceptable surface smoothness, supporting their suitability for intraoral use.

## 1. Introduction

According to the glossary of prosthetic terms, an occlusal device is defined as any removable artificial occlusal surface used for diagnostic or therapeutic purposes that affects the relationship between the mandible and maxilla [1]. Occlusal splint therapy is described as an art and science that utilizes removable appliances to achieve neuromuscular coordination in the masticatory system and to create a mechanical disadvantage against parafunctional forces [2]. Occlusal splints represent one of the primary treatment options for patients suffering from temporomandibular disorders (TMDs) and bruxism [3].

Splint therapy has been reported to have various positive effects in the management of bruxism and TMD, including ensuring occlusal stabilization, preventing tooth wear, and reducing muscle pain and tension-type headaches. In the literature, splint applications have been reported to be successful in 70–90% of TMD cases [4]. However, due to the potential for high occlusal forces to develop in patients’ mouths, the materials used in the production of occlusal splints must possess optimal mechanical properties [5].

Resin systems are the primary materials used for occlusal splints. The high prevalence of TMD and the increasing clinical popularity of occlusal splints necessitate research into new and reliable devices as well as production methods [6]. Traditionally, splints are manufactured using heat-curing polymethyl methacrylate (PMMA), vacuum-formed thermoplastic resins, computer-aided design/computer-aided manufacturing (CAD/CAM) milling, and three-dimensional (3D) printing methods [7,8].

The proliferation of CAD/CAM technologies in modern dentistry has led to the development of subtractive and additive manufacturing techniques. Additive manufacturing (3D printing), introduced in 1986, has gradually gained acceptance in the dental industry [9]. Today, stereolithography (SLA) and digital light processing (DLP) are the most used additive manufacturing techniques for occlusal splints [5,9,10,11].

SLA printers use an ultraviolet (UV) laser to cure photosensitive liquid polymers, while DLP printers use high-power LEDs and photosensitive resin materials to build layers using micromirrors that control light reflection [12,13]. The fundamental difference between the two systems lies in the light source and layer exposure method. In dental practice, DLP technology stands out due to its rapid prototyping capability and shorter printing time [5]. Also, research indicates that printing orientation influences surface properties in SLA and DLP technologies. Although both methods utilize photopolymerization, SLA cures resin in a sequential manner with a UV laser, while DLP projects entire layers at once [14]. These technological distinctions affect the depth of the polymerization process, surface quality, and the mechanical performance of the material. Print orientation has a significant effect on roughness, gloss, and flexural strength in both systems, highlighting the importance of optimizing print orientation to achieve esthetic and durable dental devices and to incorporate these factors into clinical and laboratory workflows [14,15,16].

The increasing popularity of 3D printing among prosthetics specialists is mainly due to its ability to reduce material waste, shorten working time, and provide flexibility in the production process [6,17]. Nevertheless, despite these advantages, the materials and techniques used in splint production require comprehensive re-examination [18]. Careful selection of printing parameters—such as layer thickness, printing orientation, and printing technology—is critical for success. These parameters directly affect the surface quality, mechanical strength, printing time, and post-processing outcomes of the printed objects [14,16,19].

One of the most common complications in clinical practice is splint fractures or deformation [20]. Therefore, mechanical properties such as bending strength and hardness are of great importance for ensuring durability [6,15,21]. Some researchers also suggest that hardness may serve as an indicator of wear resistance [22,23,24].

From a clinical perspective, patient comfort and satisfaction are highly dependent on the surface characteristics of the splint [25]. Surface gloss is an important aesthetic and functional factor, as glossiness decreases with increasing surface [26]. Additionally, rougher surfaces are more prone to microbial adhesion [27,28,29]. The polishability and long-term gloss retention of a splint are closely related to the hardness of the resin material used [28,30,31].

Surface roughness plays a critical role in microbial adhesion and plaque accumulation. Studies have demonstrated that bacterial colonization increases markedly when *Ra* values exceed established thresholds, which can compromise oral hygiene and lead to secondary complications [29,31,32]. Consequently, achieving smooth surfaces is necessary to reduce biofilm formation and improve patient comfort.

Anisotropy, defined as the directional dependence of material properties, is an inherent characteristic of additively manufactured polymers due to their layer-by-layer fabrication process [33,34]. This phenomenon significantly influences the mechanical performance of 3D-printed dental devices, including occlusal splints, where variations in flexural strength, wear resistance, and dimensional stability have been reported across different build orientations and layer thicknesses [35,36]. Studies indicate that printing orientation and resin composition are critical determinants of anisotropic behavior, with horizontal orientations generally improving flexural strength while vertical orientations increase susceptibility to fracture and distortion [33]. Understanding and mitigating anisotropy is essential for optimizing occlusal splint design, as these devices must withstand functional loads and maintain dimensional accuracy under clinical conditions [17].

A review of existing studies indicates that the success of 3D printing technology is primarily determined by the selected printing parameters [37,38]. Layer thickness, printing orientation, and printing technology are recognized as critical factors influencing both surface quality and mechanical performance [39,40]. While previous research has investigated individual printing parameters, studies assessing the combined effects of resin type, layer thickness, and printing orientation on the surface and mechanical properties of occlusal splints remain limited [15,41].

The present study addresses this gap by simultaneously analyzing these parameters and their interactions, thereby providing a more comprehensive understanding of their complex effects on clinical performance.

Therefore, the aim of this in vitro study is to evaluate and compare the mechanical and surface properties—such as gloss, surface roughness, microhardness, flexural strength, and elastic modulus—of three commercially available 3D printing resins used in the production of rigid occlusal splints.

The null hypothesis of this study is that there is no statistically significant difference between the gloss, surface roughness, microhardness, flexural strength, and elastic modulus values of rigid occlusal splints produced using three different 3D printing resins.

## 2. Materials and Methods

### 2.1. Specimen Preparation

**Figure 1:**
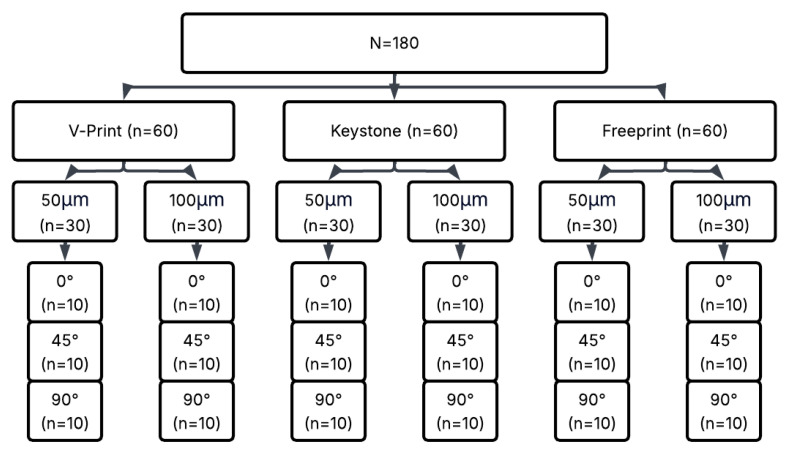
Experimental workflow of the study.

Three different commercial 3D-printing-compatible occlusal splint materials—KeySplint Hard, Freeprint Splint 2.0, and V-Print Splint—were used to prepare rectangular rod-shaped specimens measuring 3.3 × 10 × 64 mm (Table 1). For each material, a combination of two different layer thicknesses (50 µm and 100 µm) and three different printing orientations (0°, 45°, 90°) was applied, creating a total of 18 experimental groups. Each group contained 10 samples, resulting in a total of 180 samples (Figure 1).

Samples were randomly allocated to experimental groups using a computer-generated randomization list to reduce selection bias. To mitigate operator-related bias, a single experienced operator, blinded to resin type, layer thickness, and printing orientation, conducted all polishing and measurement procedures. Measurements were conducted under standardized conditions with calibrated instruments, in accordance with ISO 2813 [42] and ASTM D523 [43] standards for gloss and ISO 20795 [44] for flexural testing. Device calibration was verified before each measurement session to maintain accuracy.

**Figure 2:**
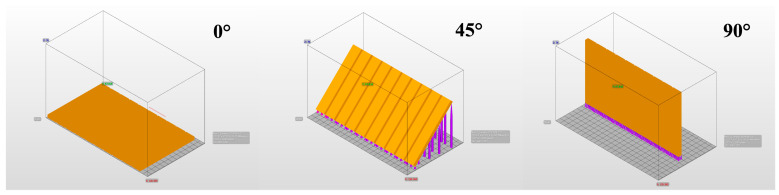
Three-dimensional designs of samples prior to printing.

The designs of the samples were prepared in STL (Stereolithography) format using 3D Builder (Microsoft, Redmond, WA, USA), and all samples were produced using an Asiga MAX UV DLP printer (Asiga, Sydney, Australia) (Figure 2). During the 3D printing process, the parameters recommended by the manufacturer were used for each resin: 385 nm wavelength, 12 mW/cm^2^ light intensity, and a curing time of 40 s per layer for KeySplint Hard; 405 nm wavelength, 10 mW/cm^2^ light intensity, and a curing time of 30 s per layer for Freeprint Splint; 385 nm wavelength, 15 mW/cm^2^ light intensity, and a curing time of 35 s per layer for V-Print Splint.

Following production, curing procedures compliant with the manufacturer’s guidelines were applied to achieve the optimal mechanical properties of the materials: KeySplint Hard was cured for 10 min in a nitrogen atmosphere using the Otoflash G171 device (BEGO, Bremen, Germany); Freeprint Splint was cured for 8 min using the DETAX Freeform cure device (DETAX GmbH & Co. KG, Ettlingen, Baden-Württemberg, Germany); V-Print Splint was cured for 10 min using the Voco Otoflash device (VOCO GmbH, Cuxhaven, Germany).

The polishing process was performed by a single experienced operator using a standardized protocol. The specimens were sequentially sanded using 600, 800, and 1200 grit sandpaper, then polished using a 3 µm diamond suspension in a Struers LaboPol-5 device (Struers, Ballerup, Denmark) at 150 rpm and 10 N pressure. To prevent operator bias, the resin type, layer thickness, and printing orientation information of the samples were concealed from the operator.

All specimens were fabricated under controlled laboratory conditions (ambient temperature: 23 ± 2 °C; relative humidity: 45 ± 5%). Distinct post-processing devices were employed for each material according to manufacturer recommendations, potentially introducing variability in polymerization efficiency and final material properties.

### 2.2. Surface Gloss

Surface gloss measurements were performed using a Landtek GM-268 gloss meter (Landtek Instruments, Guangzhou, China) in accordance with ISO 2813 [42] and ASTM D523 [43] standards. The device was calibrated with a black glass standard before each measurement session. Measurements were taken at a 60° light incidence angle from three different points on each sample, and the average values were calculated.

### 2.3. Surface Roughness

Surface roughness analysis was performed using a Mahr Marsurf M 300 C profilometer (Mahr GmbH, Göttingen, Germany). The samples were measured in two directions by rotating them 90°, the diamond tip scanned the surface with a force of 0.7 mN and a speed of 0.5 mm/s, and the cutting length was standardized to 0.25 mm and the measurement length to 2 mm. The *Ra* (arithmetic mean roughness) and *Rz* (maximum height roughness) parameters were evaluated. During the measurement process, blinding was performed by concealing the sample group information (resin type, layer thickness, printing orientation) to prevent operator bias.

### 2.4. Surface Topography

Surface topography analysis was performed using a scanning electron microscope (SEM). Samples were fixed with double-sided conductive carbon tape and sputter-coated with approximately 45 Å thickness of 80% Au–20% Pd using a Polaron SC-7620 mini sputter coater (Polaron Equipment Ltd., Watford, UK). SEM imaging was performed under low vacuum conditions using a ZEISS GEMINI 500 FESEM (Carl Zeiss AG, Oberkochen, Germany) at 100× and 250× magnification to evaluate layer morphology and surface texture. These images were used to visually corroborate profilometric findings.

### 2.5. Flexural Strength

The three-point bending test for mechanical properties was performed using the SHIMADZU AGS-X Universal testing machine (Shimadzu Corp., Kyoto, Japan) in accordance with ISO 20795 [44]. The specimens were conditioned in a water bath at 37 °C for 48 ± 2 h prior to testing, with the distance between supports set at 50 mm and the crosshead speed at 5 mm/min. Bending strength (σ) and elastic modulus (E) were calculated using the following equations: σ = 3 FL/(2b × h^2^), where F is the load (N), L is the distance between supports (mm), b is the sample width (mm), and h is the sample thickness (mm); E = (F/d) × L^3^/(4b × h^3^), where d is the deformation (mm). The modulus values were converted from MPa to GPa.

### 2.6. Surface Hardness

Surface hardness was measured using the Vickers microhardness test on a Shimadzu HMV-2 device (Shimadzu Corp., Kyoto, Japan). The average HV = 1.854 × (F/L^2^) value was calculated from three regions of each sample using a load of 200 g and a dwell time of 15 s.

### 2.7. Statistical Analysis

The sample size was calculated using the “GPower” Software (GPower 3.1.9.213, Heinrich Heine Universität Düsseldorf Institute Experimentelle Psychologie, Düsseldorf, Germany). Considering that robust ANOVA was used to determine the main and interaction effects of resin type, layer thickness, and printing orientation on surface roughness (*Ra*), gloss, microhardness, flexural strength, and elastic modulus, a medium effect size (η^2^ = 0.25), α = 0.05, and power (1–β) = 0.80 were targeted. The analysis indicated that sufficient statistical power would be achieved with 10 samples per experimental group (a total of 180 samples).

Statistical analysis was performed using Jamovi 2.3.28 software. The normality of the data was assessed using the Shapiro–Wilk test, and Robust ANOVA was used with the WALRUS package for non-normally distributed data. Bonferroni correction was applied for multiple comparisons, descriptive statistics were presented as trimmed mean ± standard error, the significance level was set at *p* < 0.05, and effect size was reported as eta-squared (η^2^).

## 3. Results

The effects of resin type, layer thickness, and printing orientation on surface characteristics (*Ra*, *Rz*, and gloss) were analyzed using two-way ANOVA (Table 2). Both resin type and printing orientation significantly affected all surface parameters (*p* < 0.001), while layer thickness showed limited influence (*p* > 0.05 for gloss, *p* < 0.001 for roughness).

Descriptive statistics are presented in Table 3. Keystone resin exhibited the lowest mean *Ra* (0.049 ± 0.003 µm), followed by Freeprint (0.126 ± 0.005 µm) and V-Print (0.194 ± 0.008 µm). Increasing layer thickness from 50 µm to 100 µm reduced surface roughness (*p* < 0.001).

**Figure 3:**
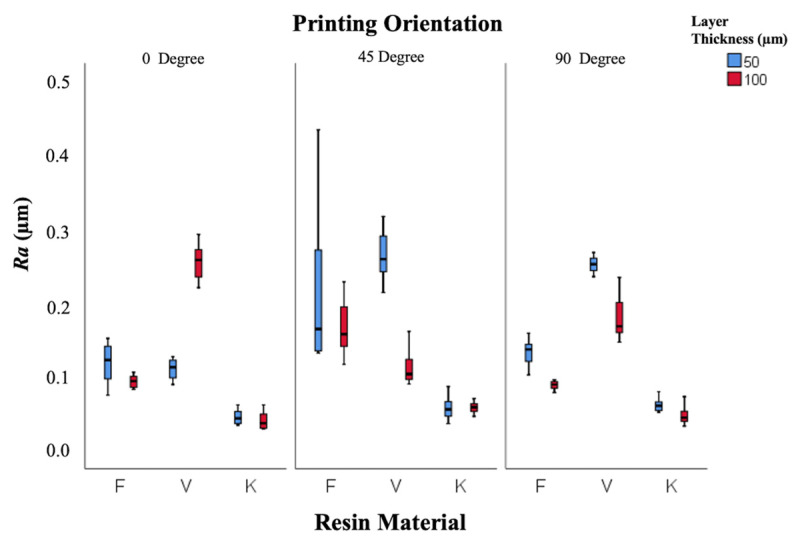
Box-and-whisker plot of roughness values according to resin, layer thickness, and printing orientation. (F: Freeprint Splint 2.0, V: V-Print Splint, K: KeySplint Hard).

As shown in Figure 3, specimens printed at 0° demonstrated smoother surfaces (*Ra* = 0.103 ± 0.003 µm) compared with those printed at 45° (0.138 ± 0.004 µm) and 90° (0.123 ± 0.004 µm).

The contact of support structures with occlusal surfaces may have indirectly influenced surface roughness and polishing performance. The highest *Ra* (0.271 ± 0.016 µm) was observed in the V-Print 50 µm–45° group, and the lowest (0.038 ± 0.004 µm) in Keystone 100 µm–90°. All *Ra* values were below the clinically acceptable 0.2 µm threshold.

**Figure 4:**
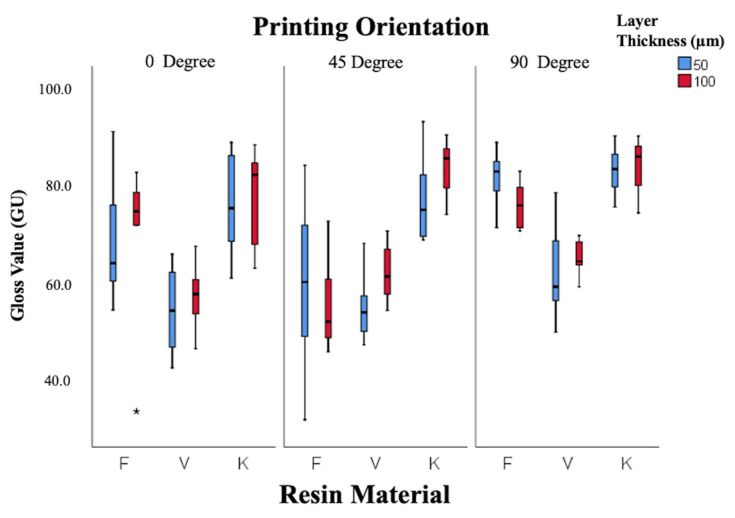
Box-and-whisker plot of gloss values according to resin, layer thickness, and angle. (F: Freeprint Splint 2.0, V: V-Print Splint, K: KeySplint Hard). (* *p* < 0.05).

Gloss values (Figure 4) were significantly affected by resin type and printing orientation (*p* < 0.001), while layer thickness showed no effect (*p* = 0.34). Keystone resin displayed the highest gloss (83 ± 1.95 GU at 100 µm, 45°), classified as excellent, whereas V-Print showed the lowest gloss (53.5 ± 3.0 GU at 50 µm, 0°), classified as poor. Gloss correlated inversely with *Ra*, confirming that smoother surfaces exhibited higher optical reflectivity.

Mechanical parameters—microhardness, flexural strength, and elastic modulus—were also influenced by the investigated variables. The ANOVA results (Table 2) revealed that both resin type and printing orientation had significant effects on microhardness (*p* < 0.001, *p* = 0.009) and elastic modulus (*p* = 0.005, *p* = 0.001), while layer thickness showed no significant influence (*p* > 0.05 for all). Mean values and standard deviations are listed in Table 3.

**Figure 5:**
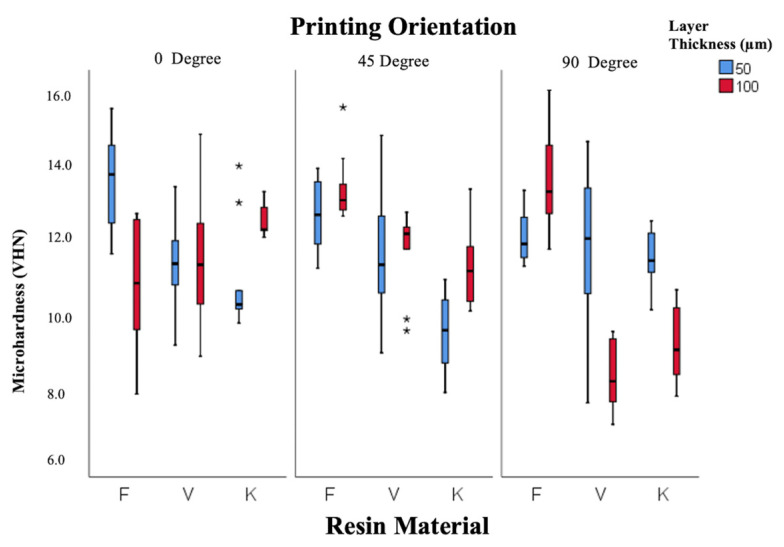
Box-and-whisker plot of microhardness values according to resin, layer thickness, and angle. (F: Freeprint Splint 2.0, V: V-Print Splint, K: KeySplint Hard). (* *p* < 0.05).

According to Figure 5, Freeprint resin showed the highest microhardness (12.6 ± 0.5 VHN), followed by V-Print (11.0 ± 0.4 VHN) and Keystone (10.6 ± 0.3 VHN). Hardness values were lower at 90° orientation compared to 0° and 45°, indicating weaker polymer cross-linking in the vertical direction.

**Figure 6:**
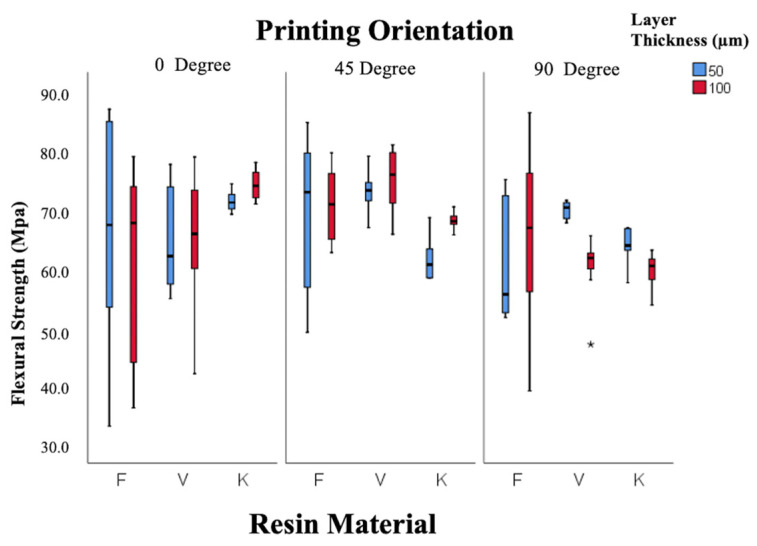
Box-and-whisker plot of flexural strength values according to resin, layer thickness, and angle. (F: Freeprint Splint 2.0, V: V-Print Splint, K: KeySplint Hard). (* *p* < 0.05).

As shown in Figure 6, flexural strength was primarily influenced by orientation (*p* = 0.001), with maximum values at 45° (69.4 ± 1.04 MPa) and minimum values at 90° (63.4 ± 1.01 MPa). Resin type and layer thickness had no significant effects (*p* = 0.150; *p* = 0.960). Some combinations, such as Freeprint 50 µm–90° and Keystone 50 µm–90°, yielded values below the ISO 65 MPa threshold [44].

**Figure 7:**
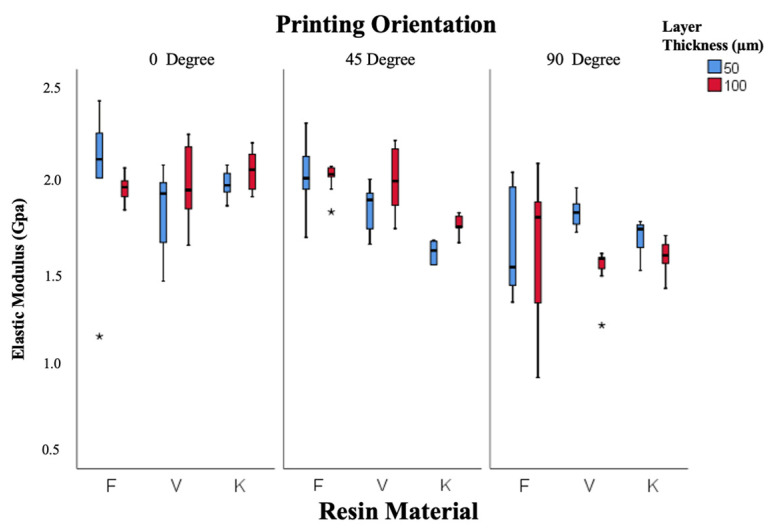
Box-and-whisker plot of elastic modulus values according to resin, layer thickness, and angle. (F: Freeprint Splint 2.0, V: V-Print Splint, K: KeySplint Hard). (* *p* < 0.05).

Elastic modulus data are illustrated in Figure 7. Both resin type and orientation were statistically significant factors (*p* = 0.005 and *p* = 0.001). Freeprint resin exhibited the highest modulus (1.89 ± 0.04 GPa), followed by V-Print (1.84 ± 0.04 GPa) and Keystone (1.76 ± 0.03 GPa). The modulus decreased as the printing orientation increased from 0° to 90°, reflecting orientation-related mechanical anisotropy. Overall, the 45° orientation produced the most balanced mechanical performance across all resins.

At 0° orientation, specimens displayed well-fused layers with minimal voids and uniform texture. At 45°, partially misaligned layer interfaces and visible step lines were observed. At 90°, distinct layer stratification and micro voids were evident, indicating weaker interlayer bonding.

A Pearson correlation analysis revealed a strong negative correlation between surface roughness (*Ra*) and gloss (r = –0.82, *p* < 0.001), indicating that smoother surfaces were associated with higher gloss values.

**Figure 8:**
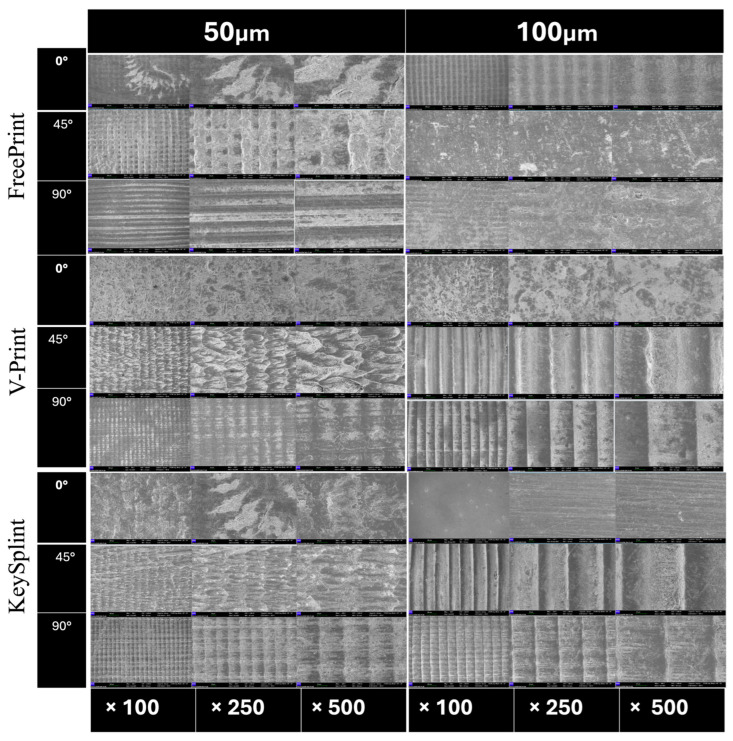
Representative SEM micrographs of splint surfaces printed at 0°, 45°, and 90° orientations with layer thicknesses of 50 µm and 100 µm, captured at 100×, 250×, and 500× magnifications. The images illustrate characteristic layer lines and resin-dependent differences in surface morphology across the three materials (Freeprint, V-Print, and KeySplint). Minor variations in image clarity—arising from inherent surface texture and resin-specific topography—do not affect the scientific interpretation of printing-orientation effects or comparative evaluations among resins.

Representative surface morphologies of the tested groups are shown in Figure 8.

These SEM findings corroborate profilometric results: smoother surfaces corresponded to lower *Ra* values and higher gloss. Keystone resin showed the most homogenous microstructure, while V-Print exhibited the most pronounced layering and interfacial irregularities. The microstructural variations observed under SEM explain both the mechanical anisotropy and surface gloss differences identified in the quantitative analyses.

## 4. Discussion

The aim of this in vitro study was to evaluate and compare the mechanical and surface properties—such as gloss, roughness, microhardness, flexural strength, and elastic modulus—of three commercially available 3D printing resins used in the fabrication of rigid occlusal splints.

According to the results, both resin type and printing orientation significantly affected several surface and mechanical properties, whereas layer thickness showed limited influence. This outcome partially rejected the null hypothesis and highlighted the complex relationship between material composition, print strategy, and post-processing conditions. These findings suggest that the mechanical and optical behavior of 3D-printed splint materials cannot be generalized, as each resin’s formulation and printing setting interact differently to determine the final performance.

When focusing on layered manufacturing, the combination of material composition, technology, printing direction, and layer thickness determines the overall mechanical behavior of printed objects [11]. Recent studies emphasize the need to optimize these parameters to improve the mechanical reliability, esthetics, and biocompatibility of printed appliances [8]. Several factors—including printing orientation, layer thickness, curing protocol, and filler content—affect not only surface smoothness but also internal stress distribution and interlayer bonding [17,20,38]. Prpić et al. [40] reported that material selection exerts a greater impact on mechanical performance than printing method alone, but that the production technology remains a key determinant. They also observed that polyamide-based, light-cured resins exhibit lower surface hardness but higher flexural strength compared with acrylic resins, possibly due to differences in filler reinforcement and the polymer cross-linking network. Consequently, assuming homogeneity across all 3D-printed splint materials could lead to misleading conclusions regarding their clinical durability and wear behavior.

In a study by Simeon et al. [15], the effect of printing orientation was found to be minimal for most resins except those fabricated by stereolithography (SLA). Wada et al. [45] also demonstrated that final curing conditions significantly influence hardness but have little effect on flexural strength across different printer types (SLA and DLP). These findings align with the current study, in which printing orientation strongly influenced surface quality, gloss, and mechanical properties, whereas layer thickness had a comparatively minor influence.

Additive manufacturing inherently introduces mechanical anisotropy due to the layer-by-layer printing mechanism and variable interlayer adhesion [33,34]. Anisotropy occurs when mechanical properties differ along build directions, which is particularly relevant for DLP and SLA resins. Wulff et al. [16] confirmed anisotropic behavior in DLP-printed splints, while other researchers found no substantial differences between orientations [19,21]. Monzon et al. [36] noted that anisotropy emerges primarily when post-curing is inadequate, while Zohdi and Yang [33] quantified approximately 5% anisotropy in DLP-printed materials. These findings suggest that the degree of post-curing and layer bonding efficiency critically determine anisotropy levels, explaining why the 45° groups in the present study demonstrated the most balanced mechanical performance.

From a clinical perspective, intraoral appliances should exhibit both sufficient rigidity and resilience to withstand occlusal forces while minimizing stress transfer to the teeth. In this regard, microhardness and elastic modulus are particularly important, as they govern resistance to wear and deformation under load [4]. Materials with lower hardness may deform over time, compromising patient comfort and occlusal stability. Polymethyl methacrylate (PMMA) has traditionally been the material of choice for occlusal splints due to its acceptable mechanical strength, optical clarity, and cost-effectiveness. However, PMMA’s drawbacks—such as polymerization shrinkage, residual monomer release, and allergenic potential—have prompted the development of digital alternatives [20,40].

Giti et al. [30] demonstrated that PMMA surfaces produced by conventional polymerization were significantly rougher than those fabricated digitally. Nevertheless, smoother 3D-printed surfaces have shown a higher affinity for Streptococcus mutans adhesion compared with conventionally fabricated ones [17]. This may be explained by variations in surface energy and hydrophobicity [30]. PMMA’s higher surface energy increases hydrophobicity, thereby reducing bacterial adhesion, while methyl methacrylate monomers can inhibit bacterial viability. Such contrasting behaviors underline that surface texture alone does not fully predict microbial colonization; chemical composition and surface energy must also be considered.

Comparative studies on splints fabricated by conventional, milled, and 3D-printed methods confirm that the manufacturing route directly affects roughness and biofilm formation [31]. Smoother surfaces tend to resist plaque accumulation, enhancing hygiene and comfort [26]. However, conventional polishing techniques may be less effective for additively manufactured resins, which possess layered surface topographies [28]. Huettig et al. [41] compared the polishability and wear resistance of splints produced by different methods and concluded that although general wear rates were comparable, additive manufacturing required improved finishing strategies. Grymak et al. [28] further emphasized that both print orientation and resin composition influence polishability and hardness outcomes.

The clinically acceptable threshold for surface roughness is 0.2 µm, as previous studies have shown that bacterial adhesion increases significantly when *Ra* exceeds this value [25,46]. Surfaces with *Ra* values below 0.2 µm are less prone to plaque accumulation, which is essential for maintaining oral hygiene and reducing the risk of secondary complications. Furthermore, smoother surfaces enhance patient comfort by minimizing irritation and enabling more effective cleaning [46]. In this study, all measured Ra values were below the 0.2 µm threshold, indicating that the tested materials achieve clinically acceptable surface quality.

The relationship between surface roughness (*Ra*) and gloss is particularly notable. Gloss is directly related to microtopography—smoother surfaces scatter less light, yielding higher gloss [25]. Therefore, both parameters are closely dependent on polishing and printing orientation. In this study, Keystone resin exhibited the highest gloss values, while V-Print displayed the lowest. This may be explained by compositional differences, including pigment content, resin transparency, and refractive index variation. Keystone’s smoother surface and higher translucency likely enhanced specular reflection, yielding superior gloss. Conversely, the microvoids and visible layer lines observed in V-Print samples under SEM may have disrupted light reflection, reducing gloss.

SEM analysis provided important structural insight supporting profilometric findings. Samples printed at 0° displayed continuous, well-fused layers and minimal voids, whereas those printed at 45° and 90° exhibited step lines and interlayer gaps. These defects can serve as stress concentrators, weakening the mechanical performance and reducing gloss. The close correlation between SEM morphology, *Ra* measurements, and optical gloss confirms that surface quality is governed by both process orientation and resin chemistry. The Keystone resin’s smoother microstructure likely explains its combination of low *Ra*, high gloss, and consistent mechanical behavior.

Flexural strength and elastic modulus results further emphasize the influence of printing orientation. The highest flexural strength observed at 45° suggests that stress distribution across inclined layers may facilitate energy dissipation and enhance bonding between successive layers. In contrast, vertically printed specimens (90°) exhibited weaker interfacial adhesion, leading to lower strength. These findings correspond with those reported by Berli et al. [5] and Perea-Lowery et al. [20], who also attributed orientation-dependent performance to interlayer cohesion and curing depth.

The elastic modulus followed a similar trend, showing significant dependence on resin type and orientation. Freeprint displayed the highest modulus values, possibly due to higher filler content or improved polymerization conversion, whereas Keystone and V-Print exhibited slightly lower stiffness. This may be explained by differences in photoinitiator systems or filler-matrix coupling, which affect the degree of cross-linking during polymerization. The modulus decreased as the orientation increased from 0° to 90°, consistent with the hypothesis that vertical specimens experience less efficient light penetration and polymer network formation.

In terms of surface hardness, Freeprint again yielded the highest values, followed by V-Print and Keystone. Hardness at 90° was generally lower, suggesting incomplete interlayer curing or weaker surface polymer cross-linking. The observed differences align with the findings of Wada et al. [45] and Wesemann et al. [23], who reported significant orientation effects on microhardness in DLP materials. These variations may also be explained by differing post-curing conditions; for instance, curing in an oxygen-rich environment may inhibit surface polymerization, while nitrogen-curing can improve hardness and modulus [45].

The relatively lower hardness values in this study compared with those reported by Berli et al. [5] and Perea-Lowery et al. [20] may be attributed to differences in printer type (DLP vs. SLA), post-curing time, or temperature. Similarly, differences in cleaning methods, solvent effectiveness, and specimen geometry can significantly influence final polymer conversion. These factors highlight the importance of standardized post-processing protocols to achieve consistent mechanical properties.

This study evaluated only static mechanical properties, although occlusal splints in clinical use are exposed to prolonged cyclic loading and fatigue. Fatigue resistance is essential for the long-term durability of intraoral appliances. Consequently, future research should employ cyclic loading protocols to more closely replicate clinical conditions. Previous studies have demonstrated that repeated loading can substantially decrease flexural strength and accelerate material degradation over time [6,16]. Therefore, while the current results offer valuable insights into initial mechanical performance, they do not fully predict long-term clinical outcomes.

In addition to technical considerations, economic and time factors should also be taken into account when optimizing printing parameters, as adjustments that improve mechanical performance may increase production time and cost, which are critical for routine clinical and laboratory workflows [13]. This study did not evaluate different layer thicknesses and printing angles in terms of time and cost. These details would be useful in terms of clinical and laboratory workflow.

Despite the controlled design, several limitations must be acknowledged. The in vitro nature of this study does not fully replicate the complex intraoral environment—saliva, thermal fluctuations, and dynamic loading could alter material performance. Only DLP technology was evaluated; comparisons with SLA or LCD techniques may yield different results due to differing light intensity and curing depth. Additionally, parameters such as water absorption, fatigue resistance, and bacterial adhesion were not investigated. Future research should incorporate long-term aging and cyclic loading tests to better simulate clinical conditions.

Furthermore, environmental factors such as ambient temperature and humidity during printing, variations in UV light source, curing duration, and post-curing atmosphere can affect material polymerization and thus mechanical properties [10,23]. Differences in washing solutions and times may also contribute to variability between studies. All these parameters should be considered when interpreting interstudy comparisons or establishing universal guidelines for 3D-printed occlusal splint fabrication.

Optimizing 3D printing parameters—particularly printing technology, resin selection, and printing orientation—can markedly improve the surface smoothness, gloss, and mechanical durability of occlusal splints. Choosing appropriate material and orientation (preferably around 45°) enhances strength and interlayer bonding, reducing the risk of fracture or premature wear. Clinically, these findings suggest that customized adjustment of printing conditions allows the production of more esthetic, durable, and patient-comfort–oriented splints suitable for long-term intraoral use.

Overall, these results reinforce that material formulation and printing orientation play decisive roles in determining surface and mechanical performance. Optimizing resin chemistry, orientation strategy, and post-curing conditions can substantially improve the smoothness, durability, and clinical performance of 3D-printed splints.

## 5. Conclusions

1. Printing orientation and resin composition had the most significant influence on both surface and mechanical properties of 3D-printed occlusal splints, while layer thickness showed only a minor effect.
2. Keystone resin demonstrated the smoothest surface and highest gloss, whereas Freeprint resin exhibited superior microhardness and elastic modulus.
3. Printing at 45° orientation generally enhanced flexural strength and maximum force, suggesting that intermediate orientations provide more balanced load distribution and improved interlayer bonding.
4. Within the tested parameters, all materials achieved clinically acceptable surface smoothness (*Ra* < 0.2 µm), indicating that properly optimized 3D printing conditions can produce durable and esthetic splints suitable for clinical use.
5. Tailoring 3D printing parameters—particularly resin selection and orientation—can enhance the performance and longevity of occlusal splints, contributing to improved patient comfort and treatment outcomes.

## Figures and Tables

**Table 1:** Details of Materials, Printers, Manufacturers, and Printing Technology.

Material	Printer	Manufacturer	Printing Technology	Chemical Composition
FreePrint Splint 2.0	ASIGA MAX^TM^ UV385	DETAX GmbH, Ettlingen, Germany	LED-based digital light processing (DLP)	Isopropylidenediphenol peg-2-dimetacrylate 90% < 95%, propenoic acid (5-ethyl-1,3-dioxon-5 yl.), diphenyl (2,4,6-thimetylbenzoyl) phosphine oxide.
KeySplint Hard	ASIGA MAX^TM^ UV385	Keystone Dental Inc., Burlington, MA, USA	LED-based digital light processing (DLP)	Based on methacrylate, photo initiator, inhibitor, pigment It was noted that detailed information about the material’s composition was not available from the manufacturer.
V-Print Splint	ASIGA MAX^TM^ UV385	VOCO (M2, Voco GmbH, Cuxhaven, Germany	LED-based digital light processing (DLP)	Polyesterdimethacrylate (50–100%) BIS-EMA (25–50%) Triethylene glycol dimethacrylate (5–10%) Hydroxypropylmethacrylate (5–10%) Diphenyl(2,4,6-trimethylbenzoyl)phosphine oxide (≤2.5%) Butylated hydroxytoluene (≤2.5%)

**Table 2:** Two-way ANOVA results showing the main effects and interactions of resin type, layer thickness, and printing orientation on surface and mechanical properties.

Parameter	Factor	F Value	*p* Value	H^2^ (Effect Size)
Ra (µm)	Resin Type	164.23	<0.001	0.869
	Layer Thickness	22.41	<0.001	0.185
	Printing Orientation	25.82	0.001	0.213
	Resin × Layer Thickness	6.12	0.005	0.071
	Resin × Printing Orientation	7.54	0.001	0.084
	Layer Thickness × Printing Orientation	5.94	0.001	0.069
	Resin × Layer × Orientation	4.33	0.001	0.052
Rz (µm)	Resin Type	151.09	<0.001	0.856
	Layer Thickness	10.27	0.002	0.108
	Printing Orientation	8.19	0.006	0.091
	Interactions	–	<0.05	–
Gloss (Gu)	Resin Type	132.46	<0.001	0.746
	Layer Thickness	1.12	0.289	0.015
	Printing Orientation	15.72	0.001	0.356
	Interactions	–	<0.05	–
Microhardness (Vhn)	Resin Type	44.91	<0.001	0.506
	Layer Thickness	3.22	0.072	0.041
	Printing Orientation	8.41	0.009	0.107
	Interactions	–	<0.05	–

Bonferroni correction applied; α = 0.05. Effect size (η^2^) is classified as small (<0.06), medium (0.06–0.14), or large (>0.14).

**Table 3:** Mean ± SD of surface (*Ra*, *Rz*, gloss, microhardness) and mechanical (flexural strength, elastic modulus) properties for all resins and printing parameters.

Property	Resin	50 µm	100 µm	0°	45°	90°
Ra (µm)	Freeprint	0.143 ± 0.013 ^A^	0.117 ± 0.011 ^B^	–	–	–
	V-Print	0.211 ± 0.017 ^C^	0.180 ± 0.015 ^D^	–	–	–
	Keystone	0.052 ± 0.003 ^E^	0.047 ± 0.003 ^E^	–	–	–
Rz (µm)	Freeprint	0.738 ± 0.060 ^A^	0.606 ± 0.051 ^B^	–	–	–
	V-Print	1.033 ± 0.087 ^C^	0.887 ± 0.075 ^D^	–	–	–
	Keystone	0.308 ± 0.019 ^E^	0.254 ± 0.016 ^E^	–	–	–
Gloss (GU)	Freeprint	69.7 ± 2.18 ^A^	67.3 ± 2.15 ^A^	–	–	–
	V-Print	56.8 ± 1.31 ^B^	61.4 ± 1.28 ^C^	–	–	–
	Keystone	78.0 ± 1.41 ^D^	81.3 ± 1.39 ^D^	–	–	–
Microhardness (VHN)	Freeprint	12.86 ± 0.26 ^A^	12.31 ± 0.30 ^A^	–	–	–
	V-Print	11.54 ± 0.29 ^B^	10.38 ± 0.38 ^C^	–	–	–
	Keystone	10.44 ± 0.31 ^BC^	10.81 ± 0.31 ^BC^	–	–	–
Max Force (N)	Freeprint	–	–	92.7 ± 6.38 ^ABCD^	101.9 ± 3.12 ^ABC^	91.5 ± 3.87 ^ACD^
	V-Print	–	–	94.7 ± 2.97 ^ACD^	107.1 ± 1.78 ^B^	95.3 ± 1.72 ^AD^
	Keystone	–	–	105.5 ± 0.89 ^BC^	92.2 ± 2.28 ^AD^	89.9 ± 1.15 ^D^
Max Elongation (mm)	Freeprint	–	–	5.62 ± 0.57 ^AB^	5.91 ± 0.32 ^A^	8.77 ± 0.42 ^CD^
	V-Print	–	–	5.85 ± 0.33 ^A^	8.68 ± 0.28 ^C^	8.68 ± 0.31 ^C^
	Keystone	–	–	7.56 ± 0.04 ^BD^	8.06 ± 0.09 ^C^	8.14 ± 0.06 ^C^
Flexural Strength (MPa)	Freeprint	–	–	63.8 ± 4.39 ^ABCD^	70.2 ± 2.15 ^ABC^	63.0 ± 2.67 ^ACD^
	V-Print	–	–	65.2 ± 2.04 ^ACD^	73.7 ± 1.23 ^B^	65.7 ± 1.18 ^AD^
	Keystone	–	–	72.7 ± 0.61 ^BC^	64.1 ± 1.57 ^AD^	61.9 ± 0.79 ^D^
Elastic Modulus (GPa)	Freeprint	–	–	2.01 ± 0.07 ^ABCD^	2.01 ± 0.04 ^ABC^	1.65 ± 0.08 ^ACD^
	V-Print	–	–	1.91 ± 0.05 ^ACD^	1.91 ± 0.04 ^B^	1.68 ± 0.04 ^AD^
	Keystone	–	–	2.01 ± 0.02 ^BC^	1.66 ± 0.04 ^AD^	1.63 ± 0.03 ^D^

Superscripts A–E indicate Bonferroni post hoc test results. Groups sharing the same letter are not significantly different (*p* < 0.05).

## Data Availability

The original contributions presented in this study are included in the article. Further inquiries can be directed to the corresponding author.
